# Assessing the Influence of Environmental Sources on the Gut Mycobiome of Tibetan Macaques

**DOI:** 10.3389/fmicb.2021.730477

**Published:** 2021-08-04

**Authors:** Binghua Sun, Yingna Xia, Samuel Davison, Andres Gomez, Paul A. Garber, Katherine R. Amato, Xiaojuan Xu, Dong-po Xia, Xi Wang, Jin-hua Li

**Affiliations:** ^1^School of Resource and Environmental Engineering, Anhui University, Hefei, China; ^2^International Collaborative Research Center for Huangshan Biodiversity and Tibetan Macaque Behavioral Ecology, Anhui University, Hefei, China; ^3^Department of Animal Science, University of Minnesota, St. Paul, MN, United States; ^4^Department of Anthropology and Program in Ecology, Evolution, and Conservation Biology, University of Illinois, Urbana, IL, United States; ^5^International Centre of Biodiversity and Primate Conservation, Dali University, Dali, China; ^6^Department of Anthropology, Northwestern University, Evanston, IL, United States; ^7^School of Life Sciences, Hefei Normal University, Hefei, China; ^8^School of Life Sciences, Anhui University, Hefei, China

**Keywords:** Tibetan macaque, gut mycobiome, environmental fungi, plant, soil

## Abstract

The distribution and availability of microbes in the environment has an important effect on the composition of the gut microbiome of wild vertebrates. However, our current knowledge of gut-environmental interactions is based principally on data from the host bacterial microbiome, rather than on links that establish how and where hosts acquire their gut mycobiome. This complex interaction needs to be clarified. Here, we explored the relationship between the gut fungal communities of Tibetan macaques (*Macaca thibetana*) and the presence of environmental (plant and soil) fungi at two study sites using the fungal internal transcribed spacer (ITS) and next generation sequencing. Our findings demonstrate that the gut, plant and soil fungal communities in their natural habitat were distinct. We found that at both study sites, the core abundant taxa and ASVs (Amplicon Sequence Variants) of Tibetan macaques’ gut mycobiome were present in environmental samples (plant, soil or both). However, the majority of these fungi were characterized by a relatively low abundance in the environment. This pattern implies that the ecology of the gut may select for diverse but rare environmental fungi. Moreover, our data indicates that the gut mycobiome of Tibetan macaques was more similar to the mycobiome of their plant diet than that present in the soil. For example, we found three abundant ASVs (*Didymella rosea*, *Cercospora*, and *Cladosporium*) that were present in the gut and on plants, but not in the soil. Our results highlight a relationship between the gut mycobiome of wild primates and environmental fungi, with plants diets possibly contributing more to seeding the macaque’s gut mycobiome than soil fungi.

## Introduction

The vertebrate gut harbors a complex microbial ecosystem, colonized by a diverse population of microbes that include bacteria, archaea, fungi, and viruses (Falony et al., 2016). These populations play a crucial role in host nutrition, immune function, development and health (Flint et al., 2012; Nicholson et al., 2012; Sommer and Bäckhed, 2013; Fung et al., 2017). Although many factors, including host genetics, physiology, behavior, diet, and group size affect the composition, structure, and stability of host gut microbiome (Linnenbrink et al., 2013; Tung et al., 2015; Suzuki et al., 2019; Zmora et al., 2019), the distribution and availability of microbes present in the host’s environment also influence gut microbial composition (Tasnim et al., 2017). Disentangling the relationship between a host’s gut microbiome and the surrounding environmental microbial pool is critical for understanding the processes of gut microbiome assembly and differences in the gut ecosystem among hosts living in different environments.

In humans and nonhuman primates, gut microbiomes are more similar among hosts sharing the same environment than among hosts living in different environments (Moeller et al., 2013; Rothschild et al., 2018; Perofsky et al., 2019). Although both the horizontal and vertical transmission of gut microbes among different hosts appear to contribute to this pattern, exposure to microbes from plants consumed, and soil, and water in a host’s local environment represent potentially important microbial seeding sources (Tasnim et al., 2017). Controlled studies of gut microbial diversity in mice found that exposure to microbes present in the soil was positively correlated with their presence of those microbes in the mouse microbiome (Zhou et al., 2016, 2018). A second study found that captive rodents (*Neotoma albigula*) fed a natural diet from the wild developed a gut bacteriome similar to wild rodents (Martinezmota et al., 2019). Similarly, Li et al. (2016) found that the gut bacterial community of wild pikas (*Ochotona curzoniae* and *Ochotona daurica*) was more similar to that of plants in their natural environment than bacterial communities in soils in their natural environment (Li et al., 2016). In this regard, Han et al. (2010) found that the composition of the gut bacteriome of grass carp (*Ctenopharyngodon idellus*) was more similar to the bacteriome present on plants they consumed than to the bacteriome in the rivers and sediment they inhabited (Han et al., 2010). Finally, source tracking between sticklebacks (*Gasterosteus aculeatus*), their environment, and their diet revealed that 13% of gut OTUs were derived from surrounding water, whereas 73% were from their prey (Smith et al., 2015).

The aforementioned studies focus on the bacterial component of the microbiome, but fungal communities, also known as the mycobiome, also play critical roles as decomposers, mutualists, and pathogens in a range of ecosystems (Tedersoo et al., 2014). In particular, many studies have reported that the fungal community in the gut (also known as the mycobiome) plays a crucial role in host nutrition and health, including altering gut bacterial composition (Huffnagle and Noverr, 2013; Getzke et al., 2019), modulating host immune responses (Wuthrich et al., 2012; Rizzetto et al., 2015), and biomass-degrading of the host diet (Solomon et al., 2016).

Less is known about the composition, function, and environmental sources of fungi in the gut. Several studies have highlighted the important role of the environment in determining a host’s gut mycobiome. For example, Barelli et al. (2020) found that the mycobiome community composition differed significantly between arboreal and ground-feeding tropical primates (*Procolobus gordonorum* and *Papio cynocephalus*) living in protected and fragmented habitats (Barelli et al., 2020). Similarly, Sun et al. (2021) reported that the diversity, composition, and functional guild of the Tibetan macaque (*Macaca thibetana*) gut mycobiome differs across populations living in different habitats (Sun et al., 2021). Remarkably, it also was found that lab mice released into a natural environment showed notable increases in gut fungi, and that the fungi isolated from rewilded mice were sufficient in increasing circulating granulocytes (Yeung et al., 2020). Finally, in the indri (*Indri indri*), a Malagasy primate, a comparison of consumed soil (geophagy) and indri faces found a pattern of 8.9% shared fungal OTUs between soil and feces (Borruso et al., 2021). However, specific and direct relationships between fungal communities in the gut of wild NHPs and the mycobiome present in their habitat remain to be clarified.

Here we aimed to better understand the associations between the gut mycobiome and the environmental mycobiome of Tibetan macaques (*Macaca thibetana*), a semi-terrestrial Near Threatened primate species endemic to China. We sequenced the gut fungal communities of two groups of free-ranging Tibetan macaques, along with the fungal communities associated with plants consumed by the macaques and soil at each field site to address two key questions. First, we compared the composition and structure of Tibetan macaque gut, plant and soil mycobiomes across study sites. Second, we tested the extent to which the composition and structure of gut mycobiome were more similar to that of local plants or soil. The results of this study will improve our understanding of the relationship between the gut fungal communities of wild primates and their habitats, including which environmental fungi are more likely to seed the Tibetan macaques’ gut.

## Materials and Methods

### Study Objects and Samples Collection

This study was carried out at two sites in southern Anhui Province, China, including Mt. Huangshan and Mt. Tianhu. More information about study sites and samples is presented in Supplementary Table 1. Mt. Huangshan has been a behavioral research and ecotourism center since 1986. The Mt. Huangshan (MH) study group is composed of 60 individuals and represents a free-ranging group that is provisioned 3 times per day with a total of 5–6 kg of corn, which is approximately 1/3 of the daily food intake of the group. Mt. Tianhu (TH) is located some 10 km from MH and the group of this site was first discovered in 2018. This group is composed of 91 macaques and has never been provisioned. Both sites share similar flora and fauna. The main diet of the MH and MT groups includes leaves, seeds, and herbs, and to a lesser extent, fruits, flowers, roots, and insects. Due to provisioning the MH group also consumes corn.

All samples were collected over a 2week period during the summer, from August 1 to 14, 2019. The monkeys were followed by our research team to ensure that each fecal sample came from a different individual. We collected the fecal sample immediately while the target individual was found to have defecated. We obtained 21 fresh fecal samples from macaques in MH and 9 fecal samples from macaques at MT. Each fecal sample represents a single individual. We also collected 13 leaf samples from 8 tree species in the home range of the MH group and 18 leaf samples from 10 tree species in the home range of the MT group. For each tree species, we collected 10 leaves from each of the two trees. Tree species information is presented in Supplementary Table 2. We selected these specific tree species because their leaves are consumed with a total relative frequency of 52% (Huffman et al., 2020). We obtained 14 topsoil samples from MH and 17 from MT. Each soil sample was a mixture of 5 individual soil cores at a depth of 0–10 cm, that were randomly sampled and selected from a 1 square meter area. Soil samples from the same site were taken 10 meters apart. We selected areas for soil samples based on the monkeys’ home range.

All fecal samples were collected and placed in a sterilized sampling tube with RNAlater (QIA-GEN, Valencia, CA). Leaves and topsoil samples were placed into a sterilized polyethylene bag as a single composite sample. All the samples were placed in ice bags and transported to the laboratory at Anhui University within 12 h of collection, and stored at −80°C. This research was approved by the Institutional Animal Care and Use Committee of the Anhui Zoological Society (permit number AHZS201711008). We performed all experiments in accordance with their approved guidelines and regulations, and complied with all principles of the China Animal Ethics Committee.

### DNA Extraction and Sequencing

Total DNA from each soil sample was extracted using the FastDNA^®^ Spin kit (Bio 101, Carlsbad, CA, United States). To avoid soil contamination, we extracted DNA from feces collected from the inner core of each fecal sample using a QIAamp^®^ Fast DNA Stool Mini kit (Qiagen). Total DNA of each plant sample was extracted using a QIAamp^®^ Fast DNA Plant Mini kit (Qiagen). DNA extraction methods were carried out according to the manufacturer’s instructions.

The total DNA extracted from the 87 samples were sent to Shanghai Majorbio Bio-pharm Technology Co., Ltd. (Shanghai, China) for sequencing. The ITS regions were identified by the ITS1F (5′-CTTGGTCATTTAGAGGAAGTAA-3′) and ITS2 (2043R) (5′-GCTGCGTTCTTCATCGATGC-3′) primers (Bokulich and Mills, 2013). PCR reaction mixtures contained 5–100 ng of DNA template, 1 × GoTaq Green master mix, 1 M MgCl_2_, and 5 pmol of each primer. Reaction conditions consisted of an initial 95°C for 2 min, followed by 35 cycles of 95°C for 30 s, 55°C for 30 s, and 72°C for 60 s, and a final extension of 72°C for 5 min. After the individual quantification step, amplicons were pooled in equal amounts, and pair-end 2 × 300 bp sequencing was performed using the Illumina Miseq platform (San Diego, CA).

### Bioinformatics and Statistical Analysis

We trimmed raw FASTQ sequencing data for the adaptor sequence and for quality control using the sliding window approach implemented in fastp (v0.19.6) (Chen et al., 2018). A window of 50 bp was set to filter the reads with a tail mass value of 20 or less. If the average mass value in the window was lower than 20, the rear bases were removed from the window, and the reads with a tail mass value of 50 bp after quality control were filtered. Those containing N bases were removed. We merged overlapping paired-end reads using FLASH (v1.2.7) (Magoč and Salzberg, 2011), with the minimum overlap set to 10 bp, the maximum error ratio of overlap area was 0.2, and the number of mismatches barcode allowed was 0. The maximum primer mismatch number was 2. Lastly, we clustered the quality-check of sequences into Amplicon Sequence Variants (ASVs) using DADA2 within Qiime 2 to truncate forward and reverse reads, to denoise the data, and to detect and remove chimeras (Callahan et al., 2016; Bolyen et al., 2019). Naive Bayes Classifier was used for taxonomic identification of the ASV sequences, and BLAST searches were conducted using Unite databases (Deshpande et al., 2016).^1^

The Shannon diversity index (Shannon), ASV richness, and unweighted and weighted UniFrac distance matrices were calculated using Qiime 2 (Bolyen et al., 2019). Plant and soil contributions to the gut mycobiome at each site were identified using Source tracker (Knights et al., 2011) within R, using default parameters. Source tracker analysis was done with and without taxonomic assignment of ASV tables produced within Qiime 2. An overall average mycobiome source contribution for both sites was also created and analyzed in Source tracker. We tested for normal distributions in alpha diversity indices, relative abundances of dominant phyla, and functional guilds using the Kolmogorov-Smirnov normality test. We used a one-way ANOVA and Tukey’s post hoc tests to test for differences across study groups in case of a normal distribution, or a Kruskal-Wallis ANOVA with Dunn’s multiple comparison test in cases of an abnormal distribution. *P-*values were adjusted using a Bonferroni correction. Principal coordinates analysis (PCoA) was performed with the R packages Made4 and Vegan.3 Permutational multivariate ANOVA (PERMANOVA) was used to test for differences in beta diversity (unweighted and weighted UniFrac distance) using the Adonis function in the vegan R package (Chen et al., 2012). Linear discriminant analysis effect size (LEfSe) was used with default options to determine the fungal taxa enriched in environmental samples (Plant and soil samples were compared independently) of each study site (Segata et al., 2011). In all analyses, the value of p was set at 0.05. We defined core abundant genera, species and ASVs as present in at least 80% of each sample type (fecal, plant, and soil) and at an average relative abundance of > 1%.

## Results

### General Patterns of the Fungal Profile

After bioinformatic processing, we obtained 7,643,987 high-quality filtered reads. To eliminate the effects of different sequencing depth on the analyses, the data set was rarefied to 32,438 sequences per sample (the minimum sequence number among 92 samples). Taxonomic assignment revealed 10 phyla, 66 classes, 168 orders, 426 families, 1,162 genera and 14,823 ASVs. Among these, the relative abundance of the unclassified fungi in the six sample groups (MH_Fecal, MT_Fecal, MH_Plant, MT_Plant, MH_Soil, MT_Soil) were 5.00, 8.69, 35.25, 36.81, 19.88 and 16.24%, respectively.

### Composition of the Gut and Environmental Mycobiome

**FIGURE 1:**
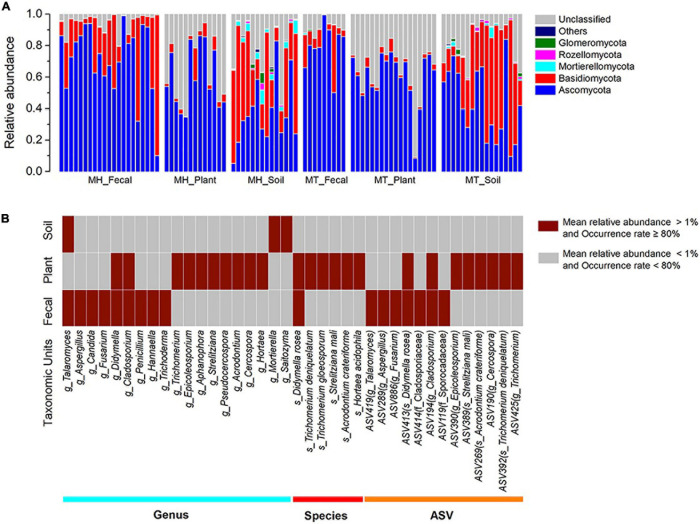
The distributions of phylum, genera, species, and ASVs. **(A)** Relative abundance of fungal taxa at the phylum level across sample groups. Stacked bar graphs illustrate the abundances of phyla and the x-axis represents the sample groups. **(B)** The distributions of core genera, species, and ASVs across sample groups. The core abundant genera, species and ASVs were defined as present in at least 80% of each sample type (fecal, plant, and soil) and at an average relative abundance of > 1%.

The dominant phyla across all samples were Ascomycota (x = mean ± Std. Deviation, Fecal: x = 73.91 ± 20.47%, Plant: x = 59.93 ± 17.65%, Soil: x = 40.54 ± 21.96%) and Basidiomycota (Fecal: x = 19.76 ± 20.98%, Plant: x = 3.91 ± 3.85%, Soil: x = 37.86 ± 24.64%) (Figure 1A). At the family level, the fecal samples were dominated by Aspergillaceae (x = 15.98 ± 16.79%) and Trichocomaceae (x = 13.85 ± 18.18%), plant samples were dominated by Trichocomaceae (x = 15.61 ± 16.87%) and Boletaceae (x = 11.11 ± 11.59%) and soil samples were dominated by Russulaceae (x = 15.34 ± 25.12%) and Aspergillacea (x = 5.51 ± 6.10%). In addition, the most common genera in the fecal samples were *Talaromyces* (x = 13.82 ± 18.50%) and *Aspergillus* (x = 9.71 ± 12.55%), whereas in the plant samples, *Trichomerium* (x = 10.86 ± 11.52%) and *Epicoleosporium* (x = 5.73 ± 12.08%) were most common. In contrast, the most prevalent fungi in the soil samples were *Russula* (x = 10.06 ± 19.40%) and *Penicillium* (x = 4.30 ± 5.50%).

The two dominant phyla, Ascomycota and Basidiomycota, showed significant variation across fecal, plant, and soil samples (Kruskal-Wallis, Ascomycota: df = 2, *F* = 29.854, *p* < 0.0001; Basidiomycota: df = 2, *F* = 51.307, *p* < 0.001) (Supplementary Figures 1A,B). A pairwise comparison analysis showed that the relative abundance of Ascomycota in fecal samples was significantly greater than those of plant and soil samples (Fecal vs. Plant: *F* = 17.385, adjusted *p* < 0.033; Fecal vs. Soil: *F* = 37.320, adjusted *p* < 0.0001; Plant vs. Soil: *F* = 19.935, adjusted *p* < 0.010). The relative abundance of Basidiomycota in fecal samples was significantly greater than those of plant samples, whereas it was significantly lower than in soil samples (Fecal vs. Plant: *F* = 29.026, adjusted *p* < 0.0001; Fecal vs. Soil: *F* = −19.232, adjusted *p* = 0.015; Plant vs. Soil: *F* = −48.258, adjusted *p* < 0.010). Linear discriminant analysis effect size analyses revealed that the plant samples of each study site was characterized by different known fungal taxa. In total, only 10 known taxa (at the genus, family, order, class, and phylum levels, and the mean relative abundance of known taxa accounting for ≥ 1% of all the plant samples) were significantly enriched in MT_Plant samples (LDA > 3, *p* < 0.05; Supplementary Figure 2A). For soil samples, 21 and 11 known taxa were significantly enriched in MH_Soil and MT_Soil, respectively (LDA > 3, *p* < 0.05; Supplementary Figure 2B).

We defined core abundant genera, species and ASVs as present in at least 80% of each sample type (fecal, plant, and soil) and at an average relative abundance of > 1%. Our results indicated the existence of seventeen core abundant taxa (9 genera, 1 species and 7 ASVs) in fecal samples, 24 (10 genera, 6 species and 8 ASVs) in the leaf samples, and three (1 genera and 2 ASVs) in the soil samples (Figure 1B). The majority core fungi abundance in the Tibetan macaque gut mycobiome were rarely present (low abundances: < 1%, low occurrence rate: < 80%) in either the leaf or soil samples. However, fecal and leaf samples shared five core abundant taxa, including two genera (g_*Didymella* and g_*Cladosporium*), one species (s_*Didymella rosea*) and two ASVs, which belong to s_*Didymella rosea* and g_*Cladosporium*. In addition, only one core abundant genus (g_*Talaromyces*) was shared by fecal and soil samples, and none was shared between leaf and soil samples. Core abundant genera, species and ASVs of fecal, plant and soil samples across two sites are presented in Supplementary Table 3.

### Diversity of the Gut and Environmental Mycobiome

We calculated the Shannon diversity index and number of ASVs observed (ASV richness) among Tibetan macaque, leaf and soil fungal communities at each site. There was no significant difference in the alpha diversity for a given sample type across the two field sites (MH_Fecal vs. MT_Fecal, MH_Plant vs. MT_Leaf, MH_Soil vs. MT_Soil), regardless of the Shannon index or ASV richness (Wilcoxon rank-sum test, *p* > 0.05). The alpha diversity showed significant variation across fecal, plant, and soil samples at Mt. Huangshan (Kruskal-Wallis, ASV richness: df = 2, *F* = 11.985, *p* = 0.002; Shannon index: df = 2, *F* = 9.050, *p* = 0.011) (Supplementary Figures 3A,B). We found that the ASV richness of leaf samples was significantly higher than that of fecal or soil samples from Mt. Huangshan (MH_Plant vs. MH_Fecal, adjusted *p* = 0.003, MH_Plant vs. MH_Fecal adjusted *p* = 0.015), whereas, only significantly difference of shannon index between MH_Plant and MH_Fecal was detected (adjusted *p* = 0.008). The similar result was detected in the Mt. Tianhu mycobiome samples (Kruskal-Wallis, ASV richness, *p* < 0.001, Shannon indexes, *p* = 0.054), except for no significantly difference of Shannon index between any two sample types (Supplementary Figures 3C,D). In addition, there was no significant difference in alpha diversity (ASV richness and Shannon index) between fecal and soil samples, regardless of at Mt. Huangshan or at Mt. Tianhu (adjusted *p* > 0.05).

**FIGURE 2:**
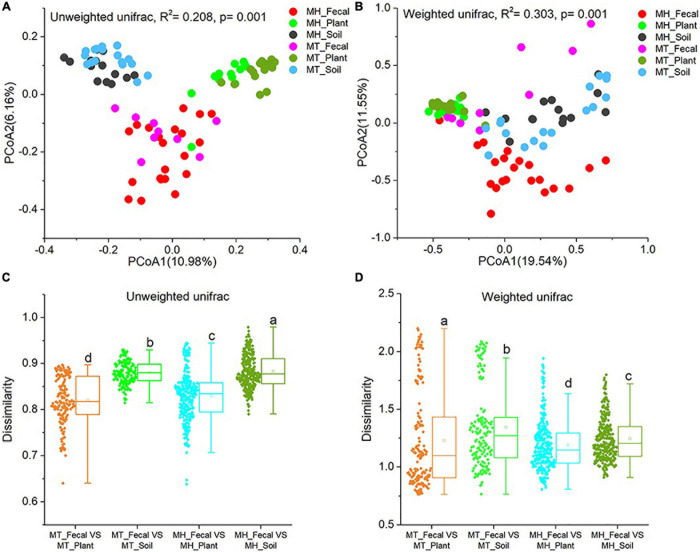
Differences in fecal fungal beta diversity across sample groups. **(A,B)** Differentiation of fecal mycobiota structure. **(A)** based on unweighted UniFrac distance, **(B)** based on weighted UniFrac distance. PCoA was used to show patterns across three study groups. Adonis tests were performed on unweighted and weighted UniFrac, respectively. **(C,D)** Comparison of dissimilarity between Gut mycobiome structure and those of environmental samples (plant and soil). **(C)** Based on unweighted UniFrac distance, **(D)** based on weighted UniFrac distance. Significance was set at the 0.05 level. Latters in **(C)** and **(D)** represnt significant diffrences between Any two sets of data.

We performed PCoA and PERMANOVA tests based on unweighted and weighted unifrac dissimilarities to investigate the variation of beta diversity in the mycobiome across all samples from the two study sites. Our result revealed significant distinctions in mycobiome profiles among sample groups (PERMANOVA, unweighted unifrac, *F* = 4.523, *R*^2^ = 0.208, *p* = 0.001; weighted unifrac, *F* = 7.486, *R*^2^ = 0.303, *p* = 0.001) (Figures 2A,B). In detail, significant differences in beta diversity between same sample types were detected based on unweighted unifrac dissimilarities (Adonis, MH_Fecal vs. MT_Fecal: *F* = 1.551, *R*^2^ = 0.053, *p* = 0.01; MH_Soil vs. MT_Soil: *F* = 2.187, *R*^2^ = 0.070, *p* = 0.001; MH_leaf vs. MT_leaf: *F* = 2.856, *R*^2^ = 0.090, *p* = 0.001). Similar result were detected based on weighted unifrac dissimilarities (Adonis, MH_Fecal vs. MT_Fecal: F = 9.36, R^2^ = 0.226, *p* = 0.001; MH_Soil vs. MT_Soil: *F* = 2.384, *R*^2^ = 0.076, *p* = 0.014; MH_Plant vs. MT_Plant: *F* = 1.981, *R*^2^ = 0.064, *p* = 0.012). In both study sites, we note that the similarity in community structure of plant and gut mycobiomes was significantly higher than that between soil and gut (Wilcoxon signed-rank test, unweighted Unifrac and weighted Unifrac, *p* < 0.001) (Figures 2C,D).

### The Shared ASVs Between Gut and Environmental Mycobiome

**FIGURE 3:**
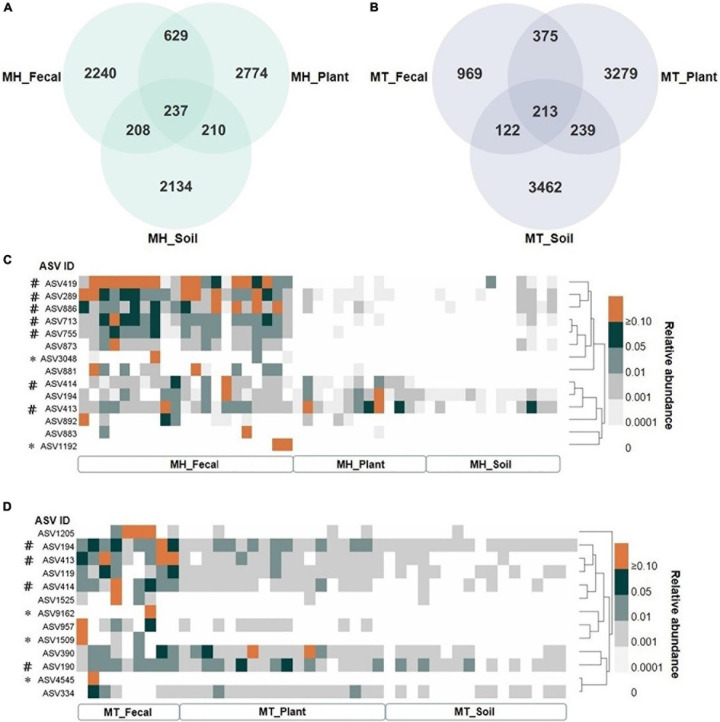
The shared ASVs between gut and environmental mycobiome. **(A,B)** The number of shared and unique ASVs among three sample types (fecal, plant and soil) at each study site, **(A)** at Mt. Huangshan (MH), **(B)** at Mt. Tianhu (MT). **(C,D)** The distribution pattern of shared ASVs across fecal, plant, and soil samples. **(C)** the dispersion pattern of shared ASVs across fecal, plant and soil samples at Mt. Huangshan (MH), **(D)** the dispersion pattern of shared ASVs across fecal, plant, and soil samples at Mt. Huangshan (MH). The core abundant ASVs were defined as present in at least 80% of each sample type (fecal, plant, and soil) and at an average relative abundance of > 1%, and the two study sites was counted separately. # Represent the core ASV of the fecal samples. * Represent the ASV in feces was not detected in both soil and fecal samples.

We identified a total of 14,823 unique Amplicon Sequence Variants (ASVs), with 4,491 in the fecal samples, 6,753 in the leaf samples, and 6,319 in the soil samples. The proportion of unique gut ASVs in Tibetan macaques at Mt. Huangshan and Mt. Tianhu were 67.80 (2,240 of 3,304 total ASVs in MH_Fecal) and 57.71% (969 of 1,679 total ASVs in MT_Fecal). There were 237 ASVs (7.15% of the total ASVs in MH_Fecal samples) shared among fecal, leaf, and soil samples at Mt. Huangshan (Figure 3A). 213 ASVs (12.7% of the total ASVs in the MT_Fecal samples) were shared among the three samples types from Mt. Tianhua (Figure 3B). In both study sites, we found that the number of ASVs shared by fecal and leaf (MH_Fecal and MH_leaf: 866 ASVs, MT_Fecal and MT_leaf: 588 ASVs) were higher than that shared by fecal and soil samples (MH_Fecal and MH_Soil: 447 ASVs, MT_Fecal, and MT_Soil: 335 ASVs).

There were 7 core ASVs at Mt. Huangshan and 4 at Mt. Tianhu (core ASV was defined as average relative abundance greater than 1 present on at least 80% of corresponding fecal samples). We found that all the core abundant ASVs present in the fecal mycobiome at each study site were present in one or both corresponding environmental samples (leaf and/or soil) (Figures 3C,D). The taxonomic profiles, mean relative abundances, and occurrence rates of these ASVs are presented in in Supplementary Tables 4, 5. The majority (>70%) of the core ASVs abundant in the Tibetan macaque gut mycobiome were rare fungal taxa (low abundances: < 1%, low occurrence rate: < 80%) in the soil and leaf environmental samples tested. However, fecal and leaf samples at Mt. Huangshan shared one core abundant ASV (*Didymella rosea*), and two ASVs (belonging to g_*Cercospora* and g_*Cladosporium)* were shared by fecal and leaf samples at Mt. Tianhu. Notably, we found that none core abundant ASV was shared by fecal and soil samples of Mt. Huangshan or Mt. Tianhu. This suggests that the connection between soil and gut mycobiome is weaker than that seen between plant and gut.

**FIGURE 4:**
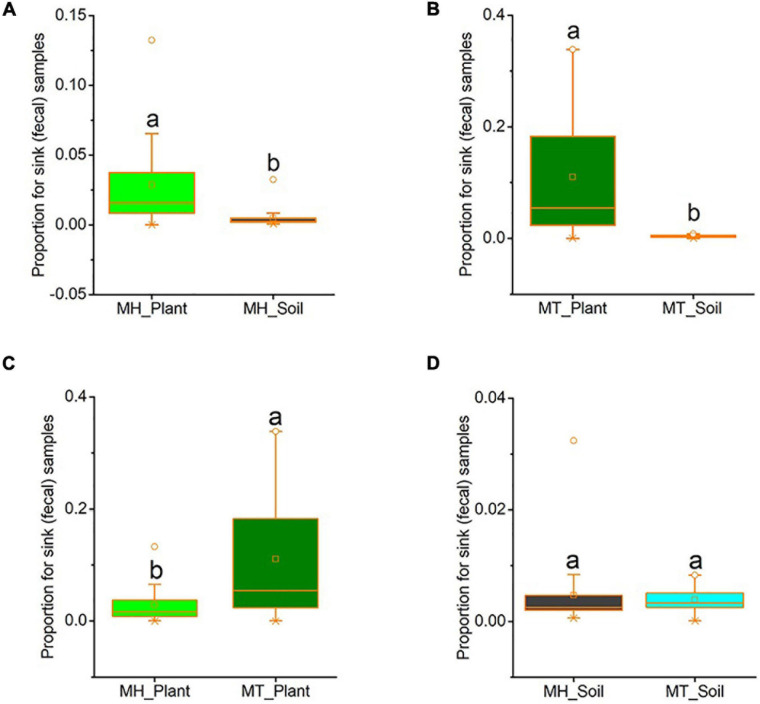
The proportion of gut fungi present in plant samples and soil samples. **(A)** Comparison between plant and soil at MH. **(B)** Comparison between plant and soil at MT. **(C)** Comparison between the two sites of plant samples. **(D)** Comparison between the two sites of soil samples. Plant and soil contributions to the gut mycobiome at each site were identified using Source tracker. Significance was set at the 0.05 level. Latters represnt significant diffrences between sample groups.

We used source tracker to estimate how much of the fecal mycobiome was from leaf or soil sources. In Mt. Huangshan, on average 2.86% of the macaques’ gut mycobiome was present in leaf sources, 0.47% were present in soil sources and 96.68% were from unknown sources. At Mt. Tianhu, on average 11.00% of the macaques’ gut mycobiome was present in leaf samples, 0.39% were present in soil samples and 88.61% were not present in either soil or leaf samples. At both sites, we found that the proportion of sink (fecal) samples from leaf sources was significantly higher than that from soil sources (Mt. Huangshan, *t* = 2.553, *p* = 0.034; Mt. Tianhu, *t* = 3.537, *p* = 0.002) (Figures 4A,B). We also found that the proportion of gut fungi present in leaf samples was lower at the Mt. Huangshan field site than from the Mt. Tianhua field site (*t* = −2.836, *p* = 0.008), no significant difference present in soil samples between the two sites (*t* = 0.351, *p* = 0.728) (Figures 4C,D).

## Discussion

We found that the two dominant known abundant phyla in Tibetan macaque guts are Ascomycota and Basidiomycota with total mean relative abundances accounting for more than 90% of the mycobiome sequences. This result is consistent with the dominant phyla observed in the guts of other mammals, such as lab mice (Wheeler et al., 2016; Yeung et al., 2020), non-human primates (Barelli et al., 2020; Borruso et al., 2021), and humans (Strati et al., 2016; Sokol et al., 2017). The dominant phyla in environmental samples (plant or soil) are also Ascomycota and Basidiomycota, although the two phyla showed significant variation in relative abundance across fecal, plant and soil sample types. In addition, we detected significant variation in mycobiome composition across all samples from the two study sites. Our results indicate that although the composition and structure of Tibetan macaque gut mycobiomes and those in plants and soil environments are obviously distinct, environmental microorganisms may have a seeding influence on the macaques mycobiome.

Whether gut fungi of the mammalian are symbionts, transient inhabitants or passengers from environments/diets is still an open question. Previous studies in humans suggested that symbiotic fungi do exist in our gut microecosystem (Fiers et al., 2019; Lai et al., 2019). However, Mann et al. (2020) reported that fungi are not natural residents of the NHPs gut and likely derive from food or environmental sources (Mann et al., 2020). In the current study, we detected seven core abundant fungal ASVs across all individuals of Tibetan macaques from the two study sites. Similar to a previous study of the pikas’ bacterial microbiome (Li et al., 2016), the majority core abundant fungal ASVs that Tibetan macaques harbor in their guts had a relatively low abundance in environmental samples. Additionally, though all the core fungi enriched in plant and soil samples could be detected in the guts of Tibetan macaques, most of them were rare fungal taxa in the guts of Tibetan macaques. This result suggests that the fungi in the gut of Tibetan macaques are unlikely to be simply a reflection of macaque environments and diets. From the wild environment to the host gut, fungi are influenced by selective pressures of the gut environment (acidic, hypoxic), host immune system, and gut bacteriome (Underhill and Iliev, 2014; Getzke et al., 2019; Richard and Sokol, 2019), which may explain why the distribution of fungi in host gut is not consistent with that in their habitat environment (plant and soil).

Studies in humans, NHPs and lab mice have shown that differences in host factors including sex, age and immune responses are associated with variation in gut mycobiome community composition (Zhang et al., 2017; Li et al., 2018; Sun et al., 2018). Furthermore, other host physical, chemical and gut bacterial factors may limit competition and invasion of foreign microbes and therefore affect colonization of environmental fungi (Sperandio et al., 2015; Sam et al., 2017). Thus, although environmental fungi may enter the gut of Tibetan macaques when they ingest plants diets or come into contact with the topsoil, they may not colonize. Further studies that involve culturomics, host immune responses to fungi, fungal adaptation to the gut niches, as well as interactions between gut bacteria and fungi, will help us determine whether these ASVs are truly gut symbionts or transient inhabitants (Fiers et al., 2019).

Previous studies found that the gut bacterial communities of pikas and grass carp were more similar to those of consumed plants than soil, water, or sediment (Han et al., 2010; Li et al., 2016). Our findings in Tibetan macaques are consistent with these reports. Independently of geographic location, the Tibetan macaque gut mycobiome was more similar to the mycobiome of dietary plant species than those of soil. Our data suggest that the gut mycobiome is assembled partly via seeding from both diet- and soil-associated fungi, with fungi in dietaryplants being the greatest contributor of the two sources. Our data showed that more than 30% of ASVs in the gut of the two wild living groups (MH: 30.20%, MT: 42.29%) was detected in their respective habitat environmental samples. Furthermore, all the core abundant ASVs of each group fecal samples could be detected in environmental samples (plant, soil or both). This finding was differing with previous study on pika gut bacterial microbiota, which reported that a majority of core ASVs in pika gut were only sporadically observed in the plant bacteriome (Li et al., 2016). In addition, the results of source-track analyses showed that the explanation of soil and diet fungi for gut mycobiome is low. The absence of others potential environmental sources may limit our full understanding in the influence of environmental sources on the Tibetan macaques’ gut mycobiome, including water, the plant species that macaques don’t eat or with low feeding frequency, as well as the corn that provision for MH group. Future studies are needed to test whether and to what extent of these environmental sources contributors to the seeding of commensal mycobiome.

Particularly, the gut of the macaques shared much more fungal ASVs with plants than soil, again implying that fungi from plants had a greater seeding effect on gut mycobiome than those from soil. Notably, gut and plant samples shared one core abundant ASV (*Didymella rosea*), and two ASVs which belong to g_*Cercospora* and g_*Cladosporium* in Mt. Huangshan and Mt. Tianhu, respectively, but none of those shared by the gut and soil. Species *Didymella rosea* and genus *Cercospora* were associated plant pathogens (*Tanacetum cinerariifolium*) (Crous et al., 2006; Pearce et al., 2016). Most species of the genera *Cladosporium* were reported as plant endophytic pathogens (Venkateswarulu et al., 2018). As Tibetan macaques are highly dependent on plants for food (Huffman et al., 2020), the plant-associated fungi may partly colonize their guts. However, it is difficult to identify if these core fungi, shared by gut and plant, are symbionts, transient inhabitants or passengers from the available data.

Furthermore, we also found that the proportion of gut fungi present in leaf samples of the Mt. Huangshan was lower than from the Mt. Tianhua. This result is likely in response to corn provision which reduced the utilization of plant leaves of individuals in Mt. Huangshan. A recent study found that Tibetan macaques translocated from the wild into a captive setting (without access to wild plant foods) for a period of 1 year, were characterized by a reduction in fungal diversity (Sun et al., 2021). Given that a reduction in fungal diversity has the potential to have negative effects on the ability of Tibetan macaques to digest cellulose, as well as their immune response (Akin et al., 1983; Denman et al., 2010; Sokol et al., 2017; Yeung et al., 2020), this shift could have implications for their health. It has been demonstrated that natural diets promote the retention of the native gut bacterial community in captive rodents (Martinezmota et al., 2019). Thus, we suggest that supplementing the diets of captive Tibetan macaques with wild plants may be a way likely to increase the diversity of the gut mycobiome. Future studies are needed to test whether this method is feasible for the restoration of the gut mycobiome of NHPs that live in captivity.

## Conclusion

Our findings demonstrate that the two most abundant phyla in Tibetan macaque guts and their surrounding environment are Ascomycota and Basidiomycota, whereas fungal community compositions and diversities of mycobiomes between Tibetan macaques and those of their living environments are distinct. Particularly, our results indicate that the gut mycobiome of Tibetan macaques is assembled partly via colonization by both plant- and soil-associated fungi, but Tibetan macaque guts appear select for diverse but rare environmental fungi. Interestingly, the current data strongly support that Tibetan macaque gut mycobiome is more similar to the mycobiome of their dietary plants than those of soil. This study does not consider other potential environmental sources (for example, water and the plant species that macaques don’t eat). In future studies, it is necessary to explore the relationship between Tibetan macaques’ gut mycobiome and environmental fungi on a broader scale.

## Data Availability Statement

The datasets presented in this study can be found in online repositories. The names of the repository/repositories and accession number(s) can be found at: NCBI, PRJNA739868.

## Author Contributions

BS and J-hL conceived and designed the experiments. BS, YX, XX, XW, and D-pX performed the experiments. BS, YX, XX, SD, PG, and AG contributed to reagents, materials, and analysis tools. BS, YX, SD, XX, PG, KA, AG, and J-hL wrote the manuscript. All authors reviewed the manuscript and agreed to the published version of the manuscript.

## Conflict of Interest

The authors declare that the research was conducted in the absence of any commercial or financial relationships that could be construed as a potential conflict of interest.

## Publisher’s Note

All claims expressed in this article are solely those of the authors and do not necessarily represent those of their affiliated organizations, or those of the publisher, the editors and the reviewers. Any product that may be evaluated in this article, or claim that may be made by its manufacturer, is not guaranteed or endorsed by the publisher.

## Footnotes

1
https://unite.ut.ee/repository.php
