# Supplementary material for: Assessing the Influence of Environmental Sources on the Gut Mycobiome of Tibetan Macaques
Source: Front Microbiol. 2021 Aug 4;12:730477. doi: 10.3389/fmicb.2021.730477 (PMC8372991; doi:10.3389/fmicb.2021.730477)
Supplement: Supplementary file 1 [file Data_Sheet_1.PDF]

Assessing the influence of environmental sources on the gut mycobiome of Tibetan Macaques - *Supplementary materials*

Binghua Sun[1, 2*], Yingna Xia[1, 2], Samuel Davison[4], Andres Gomez[4], Paul A. Garber[5], Katherine R. Amato[6], Xiaojuan Xu[2,3], Dongpo Xia[2, 7], Xi Wang[1, 2], Jinhua Li[1, 2, 3*]

1 School of Resource and Environmental Engineering, Anhui University, Hefei, China

2 International Collaborative Research Center for Huangshan Biodiversity and Tibetan Macaque Behavioral Ecology, Anhui University, Hefei, China

3 School of Life Science, Hefei Normal University, Hefei, China

4 Department of Animal Science, University of Minnesota, St Paul, MN USA 55108

5 Department of Anthropology and Program in Ecology, Evolution, and Conservation Biology, University of Illinois, Illinois, Urbana, and International Centre of Biodiversity and Primate Conservation, Dali University, Dali, Yunnan, China

6 Department of Anthropology, Northwestern University, Evanston, IL, USA, 60093

7 School of Life Science, Anhui University, Hefei, China

Table S1. The information of study sites and samples. Mt. Tianhu is about 10 kilometers away from Mt. Tianhu, all the samples were sampled period.

Study site		Main Food type	Living state	Sample type	Sample number
Full name	Abbreviation				
Mt. Huangshan	MH	wild plant and Feed corn	Free-ranging	Fecal	21
				Plant leaves	13
				topsoil	14
Mt. Tianhu	MT	wild plant	wild	Fecal	9
				Plant leaves	18
				topsoil	17

Table S2. Plant samples information. Two samples of the same species come from different plants. The relative frequency of consumption of each plant specie during our study time is from You et al. (2013).

Plant family and species	Relative frequency of consumption (*a*)	Plant part	Number	
			HS	TH
**Fagaceae**				
*Castanpopsis eyre*i (Champion ex Bentham) Tutcher	10.49%	leaf	1	3
Lithocarpus glaber (Thunb.) Nakai	4.27%	leaf		1
Quercus glauca Thunb.	5.05%	leaf	2	2
**Lauraceae**				
Litsea coreana H. Léveillé	9.55%	leaf	2	2
Machilus leptophylla Handel-Mazzetti	6.84%	leaf	2	2
**Poaceae**				
Carex tristachya Thunberg in Murray	4.78%	leaf	2	2
**Hamamelidaceae**		leaf		
Loropetalum chinense (R. Brown) Oliver	3.51%	leaf	1	1
**Theaceae**		leaf		
Camellia cuspidata (Kochs) H. J. Veitch	2.84%	leaf		2
Eurya nitida Korthals	3.58%	leaf	1	2
**Saxifragaceae**				
Itea omeiensis C. K. Schneider in Sargent	1.09%	leaf	2	1
Total	52.00%		13	18

Table S3 Core abundant genera, species and ASVs of fecal, plant and soil samples across two sites. Core taxonomy units were identified as those present on at least 80% of each sample types (fecal, plant and soil) and at an average relative abundance of >1%. [a]: mean relative abundance, [b]: Occurrence rate.

Taxonomic Level	Taxonomy Units	Fecal samples		Plant samples		Soil samples	
		M.R.A[a]	O.R[b]	M.R.A[a]	O.R[b]	M.R.A[a]	O.R[b]
Genus	g_*Talaromyces*	**0.1382**	**96.67%**	0.0005	67.74%	**0.0257**	**80.65%**
	g_*Aspergillus*	**0.0971**	**96.67%**	0.0004	70.97%	0.0042	70.97%
	g_*Candida*	**0.0714**	**96.67%**	0.0000	22.58%	0.0000	3.23%
	g_*Fusarium*	**0.0459**	**96.67%**	0.0007	80.65%	0.0084	58.06%
	g_*Didymella*	**0.0312**	**96.67%**	**0.0207**	**90.32%**	0.0035	38.71%
	g_*Cladosporium*	**0.0290**	**86.67%**	**0.0156**	**96.77%**	0.0018	93.55%
	g_*Penicillium*	**0.0194**	**96.67%**	0.0012	87.10%	0.0430	96.77%
	g_*Hannaella*	**0.0120**	**86.67%**	0.0022	90.32%	0.0012	32.26%
	g_*Trichoderma*	**0.0120**	**86.67%**	0.0004	74.19%	0.0042	96.77%
	g_*Trichomerium*	0.0080	96.67%	**0.1086**	**100.00%**	0.0008	58.06%
	g_*Epicoleosporium*	0.0078	63.33%	**0.0573**	**100.00%**	0.0004	45.16%
	g_*Aphanophora*	0.0000	20.00%	**0.0362**	**90.32%**	0.0000	3.23%
	g_*Strelitziana*	0.0040	96.67%	**0.0275**	**100.00%**	0.0003	29.03%
	g_*Pseudocercospora*	0.0013	56.67%	**0.0273**	**93.55%**	0.0012	45.16%
	g_*Acrodontium*	0.0032	70.00%	**0.0135**	**100.00%**	0.0001	32.26%
	g_*Cercospora*	0.0060	60.00%	**0.0134**	**96.77%**	0.0023	61.29%
	g_*Hortaea*	0.0005	50.00%	**0.0123**	**93.55%**	0.0000	19.35%
	g_*Mortierella*	0.0009	60.00%	0.0000	6.45%	**0.0186**	**90.32%**
	g_*Saitozyma*	0.0049	83.33%	0.0007	38.71%	**0.0141**	**87.10%**
Species	s_*Didymella rosea*	**0.0301**	**96.67%**	**0.0167**	**90.32%**	0.0023	38.71%
	s_*Trichomerium deniquelatum*	0.0033	83.33%	**0.0692**	**100.00%**	0.0002	54.84%
	s_*Trichomerium gloeosporum*	0.0028	80.00%	**0.0274**	**96.77%**	0.0001	32.26%
	s_*Strelitziana mali*	0.0025	63.33%	**0.0219**	**93.55%**	0.0002	25.81%
	s_*Acrodontium crateriforme*	0.0032	70.00%	**0.0135**	**100.00%**	0.0001	22.58%
	s_*Hortaea acidophila*	0.0005	50.00%	**0.0123**	**93.55%**	0.0000	19.35%
ASV	ASV419(g_*Talaromyces*)	**0.1304**	**90.00%**	0.0004	38.71%	0.0008	22.58%
	ASV289(g_*Aspergillus*)	**0.0605**	**90.00%**	0.0003	58.06%	0.0004	29.03%
	ASV886(g_*Fusarium*)	**0.0451**	**96.67%**	0.0004	41.94%	0.0003	22.58%
	ASV413(s_*Didymella rosea*)	**0.0301**	**96.67%**	**0.0167**	**90.32%**	0.0023	35.48%
	ASV414(f_Cladosporiaceae)	**0.0276**	**83.33%**	0.0037	77.42%	0.0002	12.90%
	ASV194(g_*Cladosporium*)	**0.0265**	**83.33%**	**0.0141**	**96.77%**	0.0016	90.32%
	ASV119(f_Sporocadaceae)	**0.0109**	**83.33%**	0.0028	96.77%	0.0013	58.06%
	ASV390(g_*Epicoleosporium*)	0.0067	63.33%	**0.0481**	**100.00%**	0.0004	41.94%
	ASV389(s_*Strelitziana mali*)	0.0020	40.00%	**0.0206**	**93.55%**	0.0002	22.58%
	ASV269(s_*Acrodontium crateriforme*)	0.0030	70.00%	**0.0135**	**100.00%**	0.0001	22.58%
	ASV190(g_*Cercospora*)	0.0058	56.67%	**0.0132**	**96.77%**	0.0022	58.06%
	ASV392(s_*Trichomerium deniquelatum*)	0.0001	20.00%	**0.0129**	**90.32%**	0.0000	12.90%
	ASV425(g_*Trichomerium*)	0.0014	73.33%	**0.0115**	**96.77%**	0.0004	19.35%

Table S4 Core abundant ASVs of fecal, plant and soil samples at Mt. Huangshan (MH). Core taxonomy units were identified as those present on at least 80% of each sample types (fecal, plant and soil) and at an average relative abundance of >1%. [a]: Sample number, [b]: mean relative abundance, [b]: Occurrence rate.

ASV ID	Taxonomy Units	MH_Fecal			MH_Plant			MH_Soil		
		S.N[a]	M.R.A[b]	O.R[c]	S.N[a]	M.R.A[b]	O.R[c]	S.N[a]	M.R.A[b]	O.R[c]
ASV419	g_*Talaromyces*	21	**0.185**	**100.00%**	13	0.000	15.38%	14	0.002	28.57%
ASV289	g_*Aspergillus*	21	**0.086**	**100.00%**	13	0.000	53.85%	14	0.001	35.71%
ASV886	g_*Fusarium*	21	**0.062**	**100.00%**	13	0.001	30.77%	14	0.000	35.71%
ASV713	f_Aspergillaceae	21	**0.038**	**95.24%**	13	0.000	15.38%	14	0.000	21.43%
ASV881	s_*Boletus erythropus*	21	0.037	47.62%	13	0.000	7.69%	14	0.000	0.00%
ASV414	f_Cladosporiaceae	21	**0.024**	**80.95%**	13	0.005	69.23%	14	0.000	0.00%
ASV413	s_*Didymella rosea*	21	**0.022**	**100.00%**	13	**0.035**	**92.31%**	14	0.004	50.00%
ASV892	f_Aspergillaceae	21	0.021	66.67%	13	0.000	23.08%	14	0.000	0.00%
ASV755	s_*Aspergillus penicillioides*	21	**0.016**	**95.24%**	13	0.000	7.69%	14	0.000	14.29%
ASV194	g_*Cladosporium*	21	0.013	76.19%	13	0.019	92.31%	14	0.001	85.71%
ASV883	g_*Gyrodon*	21	0.011	9.52%	13	0.000	7.69%	14	0.000	0.00%
ASV873	s_*Aspergillus europaeus*	21	0.011	76.19%	13	0.000	0.00%	14	0.000	21.43%
ASV1192	s_*Cladosporium velox*	21	0.042	9.52%	13	0.000	0.00%	14	0.000	0.00%
ASV3048	p_Basidiomycota	21	0.016	19.05%	13	0.000	0.00%	14	0.000	0.00%

Table S5 Core abundant ASVs of fecal, plant and soil samples Mt. Tianhua (MT). Core taxonomy units were identified as those present on at least 80% of each sample types (fecal, plant and soil) and at an average relative abundance of >1%. [a]: Sample number, [b]: mean relative abundance, [b]: Occurrence rate.

**ASV ID**	**Taxonomy Units**	**MT_Fecal**			**MT_Plant**			**MT_Soil**		
		S.N[a]	M.R.A[b]	O.R[c]	S.N[a]	M.R.A[b]	O.R[c]	S.N[a]	M.R.A[b]	O.R[c]
ASV1205	g_*Candida albicans*	9	0.190	55.56%	18	0.000	16.67%	17	0.000	5.88%
ASV194	g_*Cladospori*um	9	**0.059**	**100.00%**	18	**0.011**	**100.00%**	17	0.002	94.12%
ASV413	f_Cladosporiaceae	9	**0.049**	**88.89%**	18	0.003	88.89%	17	0.001	23.53%
ASV414	s_*Didymella rosea*	9	**0.035**	**88.89%**	18	0.003	83.33%	17	0.000	23.53%
ASV1525	g_*Botrytis*	9	0.031	44.44%	18	0.000	5.56%	17	0.000	5.88%
ASV119	f_Sporocadaceae	9	0.026	77.78%	18	0.003	100.00%	17	0.000	47.06%
ASV9162	g_*Russula*	9	0.026	11.11%	18	0.000	0.00%	17	0.000	0.00%
ASV957	g_*Batcheloromyces*	9	0.023	55.56%	18	0.000	55.56%	17	0.000	0.00%
ASV390	g_*Epicoleosporium*	9	0.020	77.78%	18	**0.032**	**100.00%**	17	0.001	47.06%
ASV1509	o_Saccharomycetales	9	0.019	33.33%	18	0.000	0.00%	17	0.000	0.00%
ASV190	g_*Cercospora*	9	**0.018**	**100.00%**	18	**0.016**	**94.44%**	17	0.004	70.59%
ASV4545	s_*Metarhizium indigoticum*	9	0.013	11.11%	18	0.000	0.00%	17	0.000	0.00%
ASV334	g_*Paraconiothyrium*	9	0.011	55.56%	18	0.006	100.00%	17	0.000	47.06%

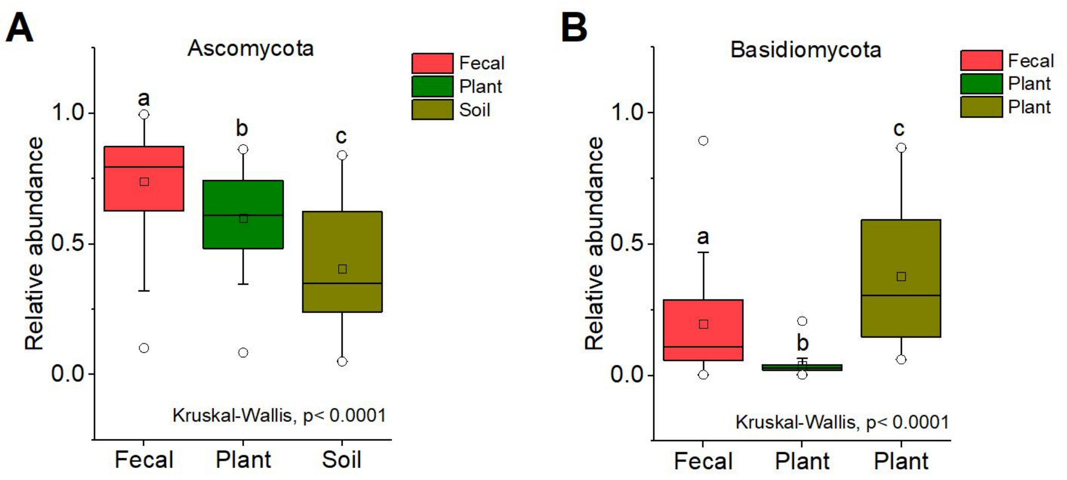

Figure S1 Differences in the dominant phylum of mycobiome across fecal, plant and soil samples. (A) Ascomycota; (B) Basidiomycota. A Kruskal-Wallis ANOVA test was used to evaluate the variation across treatment groups. Post hoc tests (Dunn's test) for pairwise comparison tests (values of p were adjusted by Bonferroni).

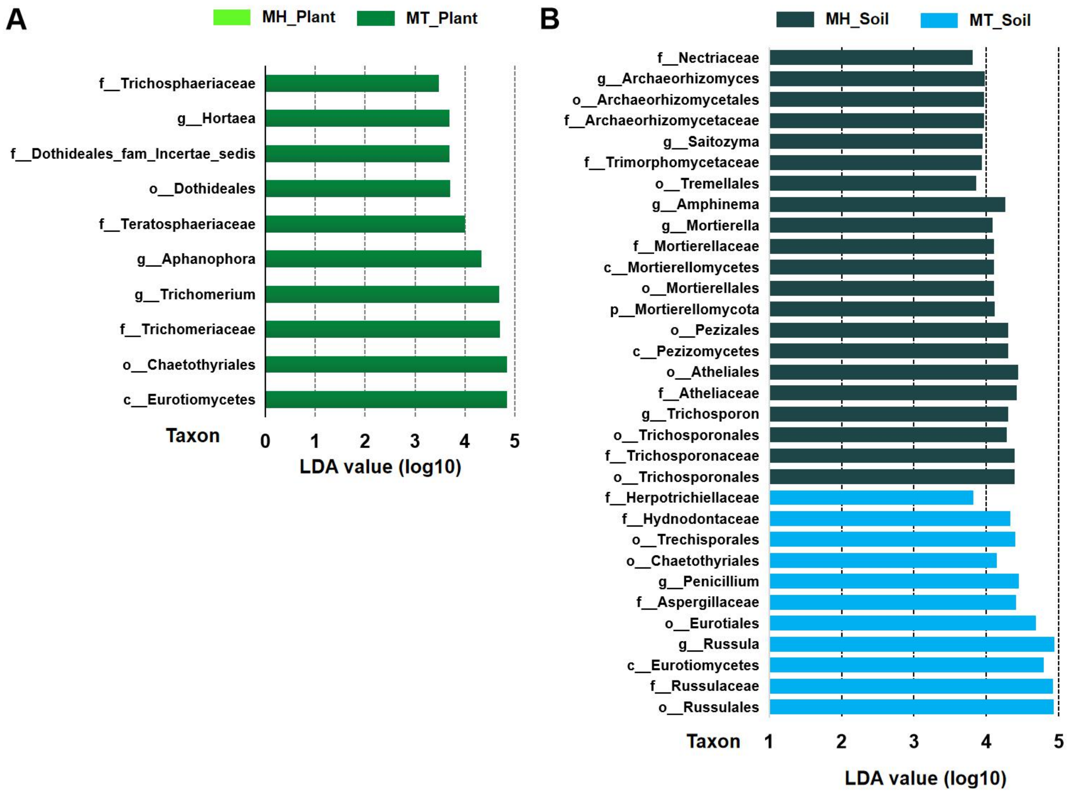

Figure S2 Indicators of known fungal taxa of environmental samples in one of the two study sites (at the genus, family, order, class, and phylum levels, the mean relative abundance of known taxa accounting for ≥1% of all the fecal sample), identified by linear discriminant analysis effect size (LEfSe) analysis (LDA > 3, $p < 0.05$). A: plant samples of the two sites (MH and MT), B: soil samples of the two sites (MH and MT).

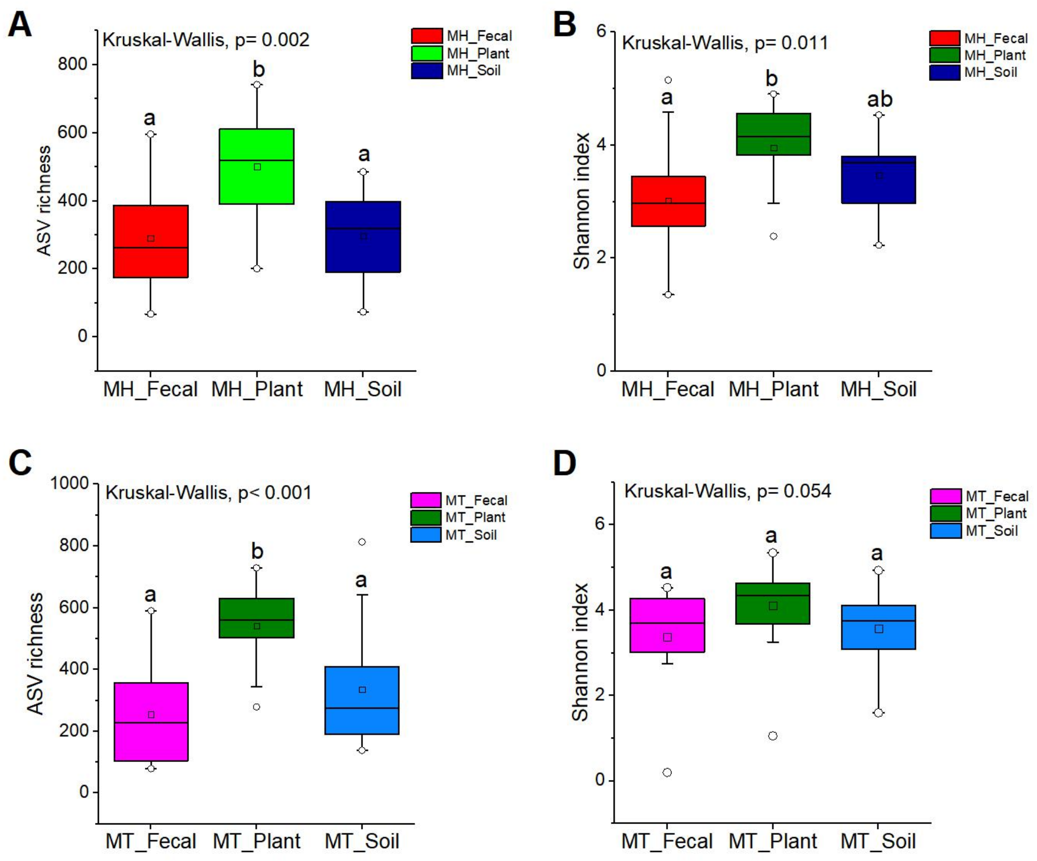

Figure S3 Differences in the Alpha diversity of mycobiome across fecal, plant and soil samples in each study sites. A, B: at Mt. Huangshan (MH), (A) ASV richness, (B) Shannon index. C, D: at Mt. Tianhu (MT), (C) ASV richness, (D) Shannon index.A Kruskal-Wallis ANOVA test was used to evaluate the variation across treatment groups. Post hoc tests (Dunn's test) for pairwise comparison tests (values of p were adjusted by Bonferroni).
